# Model Adaptation and Validation for Estimating Methane and Ammonia Emissions from Fattening Pig Houses: Effect of Manure Management System

**DOI:** 10.3390/ani14060964

**Published:** 2024-03-20

**Authors:** Paria Sefeedpari, Seyyed Hassan Pishgar-Komleh, Andre J. A. Aarnink

**Affiliations:** Wageningen Livestock Research, Wageningen University and Research, P.O. Box 135, 6700 AC Wageningen, The Netherlands; hassan.pishgarkomleh@wur.nl (S.H.P.-K.); andre.aarnink@wur.nl (A.J.A.A.)

**Keywords:** methane emission, ammonia emission, modelling, manure, pig house

## Abstract

**Simple Summary:**

Predicting emissions from livestock housing is crucial for helping farmers understand how various factors impact emissions at the pig house level. This knowledge empowers decision-makers and the farmers to make informed choices that encourage emission reduction and improve air quality in pig houses. To create innovative, low-emission pig housing systems, the development of a prediction model appears to be a straightforward solution. This paper introduces such a model for predicting methane and ammonia emissions from two commercial pig houses with two manure management systems: one with long storage in deep pits and the other with short storage, through daily flushing of a shallow pit with sloped walls (a reduced emitting surface) and partial manure dilution. This simulation model, which considers factors like animal weight, age, feed, and pen dimensions as input data, calculates manure quantity and quality, as well as methane and ammonia emissions over the growing period. In this model, the calculation of ammonia emission involves aggregating emissions from various sources identifiable within pig houses. These sources include manure channels, slatted floors, and solid floors, encompassing fouled areas, pigs, and pen partitions. This model successfully predicts the development of methane and ammonia emissions based on animal and farm characteristics.

**Abstract:**

This paper describes a model for the prediction of methane and ammonia emissions from fattening pig houses. This model was validated with continuous and discrete measurements using a reference method from two manure management systems (MMS): long storage (LS) in deep pits and short storage (SS) by daily flushing of a shallow pit with sloped walls and partial manure dilution. The average calculated methane and ammonia emissions corresponded well with the measured values. Based on the calculated and measured results, the average calculated CH_4_ emission (18.5 and 4.3 kg yr^−1^ per pig place) was in between the means from the continuous data from sensors (15.9 and 5.6 kg yr^−1^ per pig place) and the means from the discrete measurements using the reference method (22.0 and 3.1 kg yr^−1^ per pig place) for the LS and SS systems, respectively. The average calculated NH_3_ emission (2.6 and 1.4 kg yr^−1^ per pig place) corresponded well with the continuous data (2.6 and 1.2 kg yr^−1^ per pig place) and the discrete measurements using the reference method (2.7 and 1.0 kg yr^−1^ per pig place) from LS and SS, respectively. This model was able to predict the reduction potential for methane and ammonia emissions by the application of mitigation options. Furthermore, this model can be utilized as a predictive tool, enabling timely actions to be taken based on the emission prediction. The upgraded model with robust calculation rules, extensive validations, and a simplified interface can be a useful tool to assess the current situation and the impact of mitigation measures at the farm level.

## 1. Introduction

Emissions of methane (CH_4_) and ammonia (NH_3_) from agriculture have profound effects on climate change, air quality, and ecosystem health [1,2,3]. CH_4_ is the second-most abundant anthropogenic greenhouse gas [4], arising preliminarily from animal digestion and manure storages [5]. In the Netherlands, livestock farming contributes to 75% of the CH_4_ emission. In pig farming, most of the CH_4_ (more than 80%) comes from the stored slurry (i.e., faeces and urine) in the pig houses. This is mainly caused by the long storage of slurry underneath the floor in the barns and from outside manure storage places [6]. An additional portion is emitted through the digestive system of pigs, particularly in the hindgut, contributing to enteric methane formation [7]. Therefore, reducing methane emission is crucial for livestock farming to meet the climate goals.

High NH_3_ concentration is detrimental for a healthy indoor climate; its emission contributes to secondary particulate matter formation and leads to excessive nitrogen deposition in nature areas [2,8,9]. Worldwide, the primary source of NH_3_ emission in agriculture originates from livestock housing, specifically the excretion of urine by livestock (Aarnink, 1993 [10,11,12]). Waste streams of the livestock sector contribute to 39% of the global NH_3_ emission [13]. In Europe, around 80% of NH_3_ production is attributed to livestock facilities [9], with pig production contributing to about 15% of the total. In the Netherlands, agriculture is responsible for 88% of NH_3_ emission released into the air [6,14], with pig farming contributing to 20% of the NH_3_ emission. The Dutch agricultural sector has marked a two-thirds reduction in NH_3_ emission since 1990 due to various mitigating measures, including a ban on the application of manure to the land between September and February, the use of low-emission housing, covering outside manure stores, and diets with reduced protein [15]. This illustrates the ongoing importance of implementing additional measures to further minimize ammonia emission from pig production facilities.

The intensification of pig farming and the transition to indoor liquid manure storage have underscored the importance of precisely estimating CH_4_ and NH_3_ emissions influenced by the manure management system (MMS). This is essential for effectively addressing environmental challenges [16]. In recent years, efforts have been made to reduce CH_4_ and NH_3_ emissions from pig houses, leading to the development of low-emission housing systems [5]. One effective approach to reducing these emissions is frequent daily removal of manure by flushing the pit beneath the slatted floor [17,18,19,20,21]. This measure can be implemented in various forms and at different levels on farms.

In livestock production systems, the primary origin of NH_3_ is the rapid hydrolyzation of urea in urine facilitated by the faecal enzyme urease and the breakdown of undigested proteins in manure [3,22]. The latter is a minor source of ammonia emission. This hydrolyzation process results in the formation of ammonium in an aqueous medium, in which the total ammoniacal N exists in equilibrium between the ionized NH_4_+ and the unionized NH_3_ forms [23]. This equilibrium is affected by temperature and pH, and higher levels cause an increased concentration of the unionized NH_3_ form [24]. When the pH is below 7, nearly all ammoniacal N exists in its ionized form, while pH values above 7 lead to a significant rise in the unionized fraction [3].

The accurate quantification and understanding of methane and ammonia emissions from the livestock sector are crucial for assessing environmental impacts, developing sustainable farming practices, and addressing air and water quality concerns; however, evaluating the impact of numerous variants on emissions is a resource-intensive endeavour, both in terms of time and cost. Direct measurements at the housing level are time- and cost-consuming; these measured methods do not readily reflect emissions from various sources [19,25]. Models and algorithms with different levels of complexity have been developed for predicting CH_4_ and NH_3_ emissions [11,26,27,28]. In contrast, many other models tend to be static and empirical [29,30], often neglecting crucial aspects of animal metabolism, such as growth composition [31]. Moreover, empirical models have not been evaluated against experimental data [32]. To mitigate these challenges, a cost-effective alternative to direct measurements is to employ predictive models that allow the assessment of various interventions at the farm level while considering the factors affecting emissions release. A mathematical static model (MESPRO model) was developed to quantify the effect of practical measures (feed intake, diet composition, manure storage time, ambient and slurry temperature) on the characteristics and amount of slurry exertion from fattening pigs [31]. Subsequently, this model was developed into a dynamic model (ANIPRO model) to simulate the effect of ammonia volatilization in pig houses with partially slatted floors for fattening pigs [33]. Their model integrated aspects such as animal metabolism, diet composition, and the quantity and quality of urine and faeces, as well as the physical, chemical, and biological processes occurring in the urine pools on the floor and in the manure pit to predict ammonia emission. While the models were initially tested for fattening pigs with measured levels of ammonia emission, in 2018, they were extended to include other pig categories and were validated for the effect of various feeding measures on ammonia emission from fattening pigs, weaned piglets, and dry and pregnant sows [34]. However, these models were not previously tested for predicting CH_4_ and NH_3_ emissions affected by emission reduction measures related to manure management. Furthermore, it was observed that dynamically predicting methane emission influenced by frequent manure removal from indoor slurry pits in pig houses holds considerable potential for emission reduction. Therefore, the objectives of this study were to enhance the existing models by incorporating a dynamic methane prediction module and validate the model for the effect of pen design and manure management on methane and ammonia emissions from fattening pig houses.

## 2. Materials and Methods

### 2.1. Global Description of the Model

This existing model approach follows a mechanistic framework, striving to capture underlying processes as accurately as possible. The prediction model consists of three primary calculation modules: (1) the excretion model (MESPRO), (2) the ammonia emission model (ANIPRO), and (3) the methane emission model developed as part of this study. The model was developed and executed using MATLAB (R2018a). The model retrieves input data from Excel files. Within the main script, specific calculation rules are established to extract the respective data of each target scenario (for instance the pig housing types) separately, as delineated in the input data files. The simulations are conducted with daily time resolution over the course of a growing period (GP). Model calculations can be used for various pig categories, housing systems, and manure pit designs. The key calculation rules and the additions to the existing model are summarized here, while detailed descriptions can be found in the Supplementary Materials (SM) file.

**Figure 1:**
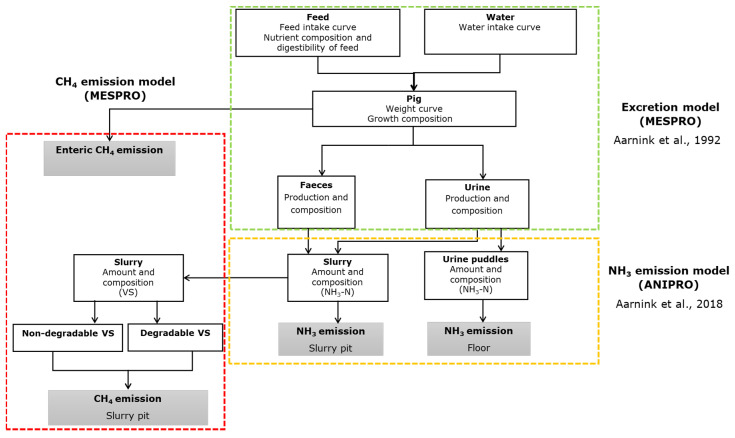
Schematic representation of the model concept for predicting ammonia and methane emissions (adopted from [31,34]).

The input variables of the model are length of production period, initial and final weight of pigs, total feed and water intake, feed composition, water use, weather data (temperature and relative humidity), climate set-up, building specifications, and storage time. The outputs, among others, are animal performance (animal weight, feed, and drinking water intake of each day), manure quantity and composition, and methane and ammonia emissions. The pH of slurry bulk was predicted based on measured data as a function of slurry total ammoniacal nitrogen, total inorganic carbon, and acetic acid concentrations (Aarnink et al., 2018 [34]). Furthermore, for a subset of parameters, calibration was executed using measurement data, i.e., the value of a parameter (e.g., a regression coefficient) was estimated based on the best fit on the measured data. This applies to three of the four parameters to estimate the Gompertz curves for animal weight and the cumulative feed and water intake (Section 2.2 of this study), the regression coefficient to calculate the evaporation coefficient for the floor and manure pit, calculation of NH_4_-N from the total N-urinary excretion, surface temperature, air velocity of the top layer of manure and of the urine puddle on the floor, and the surface pH using regression lines based on lab measurements. The calculation rules have been extensively described in previous studies by [31,34]. A schematic representation of the model is shown in Figure 1.

### 2.2. MESPRO Module

As depicted in Figure 1, the model calculates both the quantity and composition of the slurry at the time of excretion and throughout the storage period within the pig house. To determine the emission on a daily basis and over the entire production period, calculation rules were established initially, focusing on estimating pig growth, feed consumption, and water intake. The patterns of growth, feed, and drinking water intake are described by the so-called Gompertz curve. Gompertz curve is a mathematical model used to describe the growth curves of animals including pigs [35,36]. This function is characterized by an initial exponential increase in weight, followed by a gradually decreasing rate of growth, eventually stabilizing at the final adult weight. The Gompertz curve is typically represented as an S-shaped curve (Equation (1)).

(1) $$W_{t}=A+W_{m}.\exp\;\left\{-exp\left[-B\left(t-t^{*}\right)\right]\right\}/1000$$

where *W_t_* represents the weight of the animal at time *t* (kg), *A* is a constant that represents a specific weight (kg), *W_m_* is the final adult weight of the animal (kg), *B* is a parameter that influences the shape of the growth curve and determines the speed of reaching the final weight (day^−1^), *t* is the age of the animal (day), t* represents the time when growth is maximal (day), 1000 is a conversion factor to convert from grams to kilograms.

The same Gompertz curve was employedto characterize the trajectory of both feed and drinking water intake over the growing period of the pigs. Parameters of the model (*A*, *B* and t*) were determined using a regression analysis on the measured data. The fourth parameter (*W_m_* in Equation (1), and its substitutes FI_m_ (total feed intake) and DWI_m_ (total drinking water intake) in feed and water intake curves, respectively) were calculated based on the measured values of the start and end weight and the total feed and water intakes during the growth period. The estimated final weight of the adult pigs was found to be a rough approximation; therefore, this parameter was assigned as variable in our calculation tool, while the other values were fixed at the values shown in Table 1. By making one parameter variable, it was possible to fit the curve to the achieved final weight, which is an input in our model. Incorporating these curves into the calculation tool and using the final weight, total feed intake, and water intake as input data enabled the estimation of parameters associated with the asymptotic values of these variables in the calculation model.

The daily feed and water intake over the entire production period was used to determine the quantity and composition of excreted manure. Furthermore, considering the production stage of the pigs, the uptake of metabolizable energy, and the (calculated) growth curve, the nutrient retention in the animals was determined. Utilizing the digestion coefficients of the feed (particularly protein) and the retention of nutrients, the quantity and quality of manure was calculated. Additionally, employing a water balance, the concentration of the nutrients was calculated [31,34].

To determine the volume of slurry discharged into the manure pit, the calculation process was initiated by estimating the combined excretion of slurry and water. The total slurry production was determined based on the excretion of dry matter (DM_exc_), after accounting for the gas produced from the hydrolysis of organic matter (biogas) and the CO_2_ formed by the hydrolysis of urea (Urea_CO2_). The total dry matter was estimated by combining the undigested organic matter (ash) with the organic matter (OM_exc_) excreted through urine and the difference between inorganic matter (ash) intake and ash excretion.

### 2.3. ANIPRO Module

Within this model, ammonia emission is calculated by summing up the ammonia emission from different sources that can be distinguished within a pig pen, including manure channels, slatted floors, solid floors (which account for fouled pigs and pen partitions), and other relevant sources. The model incorporates physical attributes and indoor climate conditions of pig houses (Equation (2)). The ammonia emission from the different sources (floor and manure pit) in pig houses is calculated using the following formula [33]:

(2) $$E_{NH3}=\frac{k_{NH3}\;\cdot A\;\cdot f\;\cdot \left[NH_{4}N\right]}{H}$$

where *E_NH_*_3_ is the ammonia emission (mol/s); *k* is the mass transfer coefficient, which is related to the air velocity and temperature of the emitting surface (m/s); *f* is the fraction of unionized ammonia in the solution, which is influenced by *pH* and temperature (*T*) and is a dimensionless value; [*NH*_4_*N*] is the total ammoniacal nitrogen concentration (mol/m^3^); *H* is Henry’s constant, which is also temperature-dependent and a dimensionless constant. By multiplying the calculated source strengths (emission per square meter) by the emissive surface (*A*), which can represent either the floor (urine puddles) or the manure pit (m^2^), the ammonia emission from each source and the total ammonia emission of the entire pig house are determined. The emitting pit surface is calculated based on the shape and dimension of the manure pit or the floor. The emitting surface of the manure pit with straight walls is equal to the dimensions of the manure pit (length × width). In a manure pit with sloping walls, the emitting surface is influenced by the height of the manure. This height can be calculated from the manure production of the pigs and the shape of the manure pit. In this model, a distinction is made between soiling of the solid floor, soiling of the concrete slatted floor (at the front of the pen), and soiling of the metal, triangular slatted floor (at the back of the pen). By multiplying the calculated source strengths (emission per square meter) by the respective surface areas, both the total ammonia emission and the ammonia emission from each emitting surface can be determined individually [34]. Additional information for the estimation of the parameters of Equation (2) are listed in the SM file (Equations (S1)–(S9)).

### 2.4. Extended MESPRO Module

The MESPRO module constitutes calculation rules for the prediction of biogas production as a result of anaerobic conditions in manure pits. Methane formation is influenced by various factors, including manure temperature, storage duration, organic matter content of the manure, and the residual aged manure in the storage acting as an inoculum [28]. In this module, CH_4_ emission from liquid manure is calculated using an algorithm proposed by [29]. This model is based on the Arrhenius equation and uses the residual volatile solids (VS) and a temperature response function for methanogenesis (Equation (3)).

(3) $$F_{t}=\left(VS_{d}+0.01VS_{nd}\right)\;e^{\left(lnA-\frac{E_{a}}{R\;T}\right)}$$

where *F_t_* is specific methane production rate (g CH_4_ kg VS^−1^ h^−1^), *VS_d_* and *VS_nd_* represent the fast and slowly degradable fractions of volatile solids (VS) (kg kg^−1^ VS), *A* is the Arrhenius parameter (g CH_4_ kg VS^−1^ h^−1^), *E_a_* is the activation energy (kJ mol^−1^), *R* is the gas constant (kJ mol^−1^ K^−1^), and *T* is temperature (K). The algorithms used to calculate the production of VS in manure and the corrected methane emission rate for the in situ temperature are presented in the SM file (Equations (S10)–(S14)). The model parameters (*E_a_*, *lnA*, *VS_d_*) utilized in this study are presented in Table 2.

In order to predict the CH_4_ emission per day of the growing period, the model uses the amount of residual manure in proportion to the amount of discharge from the inside pit to an outside destination. On a daily time step, the loading of actual manure level inside the pig house is simulated according to the initial height of manure at the beginning of the growing period, removal rate, and the last day of emptying the pit (day of the growing period). In other words, the model first estimates the composition of the remaining manure in the pit and subsequently simulates the methane production rates per day of storage in the manure pit underneath the slatted floor. Thus, the model assumes a linear relationship between the volume of manure and CH_4_ emissions.

### 2.5. Model Application

**Figure 2:**
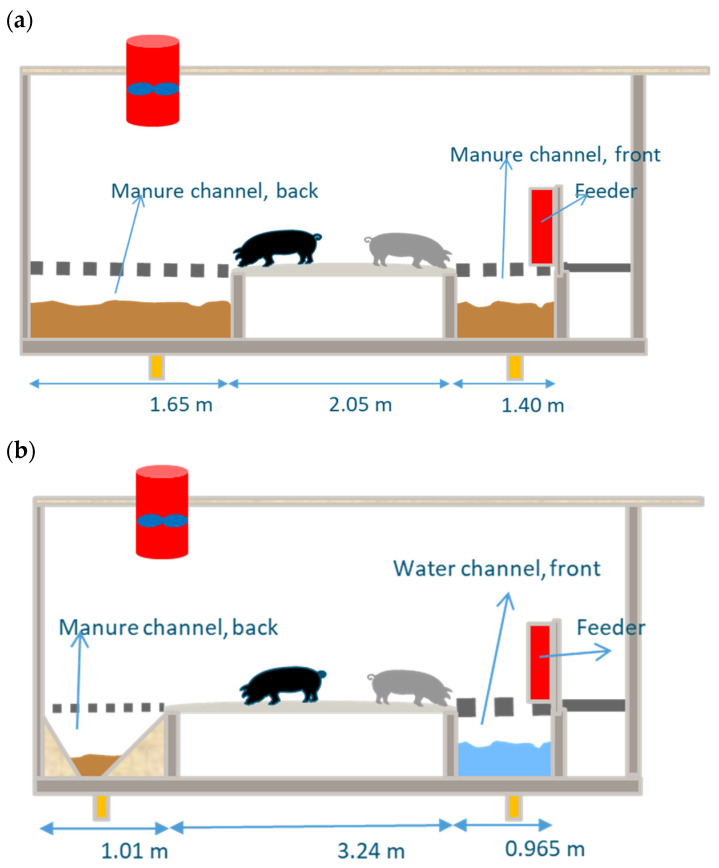
Schematic representation of the fattening pig houses (**a**) long-term storage (LS) and (**b**) short-term storage (SS) of manure inside the pig house. In the LS system, both front and back channels were filled with manure, while in the SS system, the back and front channels were filled with manure and manure diluted with water (cleaning water and spoiled drinking water).

To show the capacity of the model to predict NH_3_ and CH_4_ emissions from commercial pig houses, we compared model simulations to measured data. Experiments were carried out in two commercial fattening pig facilities over one year, from October 2020 to October 2021, including four growing periods, each lasting around 90 days. These experiments were conducted in two separate rooms, each representing a distinct manure management system (MMS). The first experimental room represented a conventional MMS with long-term storage (LS system) of manure in deep pit underneath the (partly) slatted floor. The second experimental room was equipped with an adapted slurry pit for short-term storage (SS system) of manure by daily flushing from the pit underneath the (partly) slatted floor, dilution of the front channel, and reduced manure-emitting surface (Figure 2). These two systems were examined for the mitigation potential of methane and ammonia emissions affected by the manure removal frequency, pen design, and dilution of manure with water.

The measured data comprised continuous readings of NH_3_ and CH_4_ concentrations (using sensors), ventilation rates, and temperature. Discrete measurements included manure production, manure composition, pen fouling (with urine), the percentage of the solid floor and the sloping walls that is moistened with urine, manure temperature, and height in the pit. Measurements for determining the emission of methane and ammonia gases were conducted using a reference method [38,39] at specific times (two randomly selected days per growing period) over one year (October 2020–2021) [40]. The characteristics of the farms and a schematic representation of the housing systems are presented in Table 3 and Figure 2.

The ammonia concentration of the outgoing air was measured continuously using a Dräger Polytron C300 sensor (Dräger, Lübeck, Germany). The data could be read remotely. The methane and carbon dioxide concentrations in the ventilation air of the departments were continuously measured by means of an ABB monitor (ABB-Uras26, ABB, Frankfurt, Germany). The outgoing concentrations were measured hourly for both LS and SS systems. The data was then written to a data-logging system (CR1000X; Campbell Scientific Inc., Logan, UT, USA), which could be read remotely.

The pig houses were mechanically ventilated by means of an automatically controlled climate computer. The actual ventilation flow was then measured with a measuring fan in the ventilation shaft with the same diameter as the fan. Each revolution of the measuring fan gave a number of pulses, and this number was continuously logged by the climate computer of the pig farm. Temperature (°C) and relative humidity (%) were measured in each department near the ventilation shafts by means of a sensor (Vaisala HMP60; Vaisala GmbH, Hamburg, Germany). These data were stored on a data-logging system (CR1000X; Campbell Scientific Inc., Logan, UT, USA).

### 2.6. Data Analysis

The CH_4_ and NH_3_ emissions were calculated from the measured concentration and ventilation rate according to a reference method [38,39]. For both pig rooms (*j* = 1, 2), the emission, *E_ij_* (kg yr^−1^ per pig place), was calculated per measurement day using the concentration of CH_4_ and NH_3_ in the outgoing air (*C_outij_*) and the incoming air *C_inij_*, (both in mg m^−3^), the average ventilation flow *V_ij_* (m^3^ h^−1^ per pig place), and the density of the gas (*ρ*) to convert the concentration (ppm) to mg m^−3^ (0.667 and 0.71 kg m^−3^ for CH_4_ and NH_3_). This was then multiplied by 24 and 365 and divided by 10^6^ to calculate the kg of methane and ammonia emissions per pig place per year (Equation (4)). For the reference measurement method, a vacancy factor of 3% was used to calculate the annual emission associated with the production cycles) [41].

(4) $$E_{ij}=(C_{{outt}_{ij}}-C_{{in}_{ij}})\;\cdot \;\rho \;\cdot \;V_{ij\;}\cdot 24\;\cdot 365/10^{6}$$

### 2.7. Model Validation

To assess the agreement between predicted and measured values and the accuracy of the model, the Mean Absolute Error (MAE) and Root Mean Square Error (RMSE) were calculated. Additionally, Y = X graphs were constructed, and the coefficient of determination (R^2^) was provided. Lower values of MAE and RMSE imply higher agreement between the predicted and measured values. However, higher values of R^2^ show a stronger correlation between predicted and measured values. The MAE, RMSE; and R^2^ were calculated using the following equations (Equations (5)–(7)).

(5) $$\mathrm{MAE}=\frac{\sum_{1}^{n}\;\left|V-\hat{V}\;\right|}{n}$$

(6) $$\mathrm{RMSE}=\frac{\sqrt{\sum_{1}^{n}\;{\left(V-\hat{V}\right)}^{2}}}{n}$$

(7) $$R^{2}=1-\frac{\sum_{1}^{n}\;{\left(V-\hat{V}\right)}^{2}}{\sum_{1}^{n}{\left(V-\bar{V}\right)}^{2}}$$

where MAE is the mean absolute error, RMSE is the root mean square error (both in unit of the parameter), *V* is the measured value, $\hat{V}$ is the predicted value, *n* is the number of values, R^2^ is the coefficient of determination, and $\bar{V}$ is the mean of the measured values.

## 3. Results and Discussion

### 3.1. Indoor Climate Parameters

A summary of the calculated and measured room temperature, manure temperature, and relative humidity for the LS and SS systems are presented in Table 4. The trend of the calculated and continuous-measured values of these parameters over one year are visualised in Figure S1 in the SM file. Based on the obtained results, the average predicted temperature was comparable with the calculated values for the two examined systems (MAE of 1.5 and 1.1 °C, RMSE of 4.0 and 3.8 °C, and R^2^ of 0.8 and 0.92 for the LS and SS systems, respectively). The mean predicted room temperatures were 22.3 °C for LS and 21.4 °C for SS, corresponding with measured values of 23.0 °C and 21.0 °C, respectively. The average temperature of the manure was measured as 24.5 °C and 22.3 °C, respectively. The lower temperature ranges for the SS system can be explained by the short storage courses and daily removal of manure in this system. The manure temperature was underestimated by the model (MAE of 6.7 and 6.8 °C, RMSE of 6.5 and 7.5 °C, and an extremely low R^2^ (<1) for the LS and SS systems, respectively). The manure temperature was calculated by using empirical relationships with the measured outgoing air temperature. This significant difference in manure temperatures suggests the need to consider temperature variation at inside storage facilities in countries such as the Netherlands, where long-term manure storage inside the pig houses is a common practice. Furthermore, the high variation between the measured and predicted temperature of manure can be due to issues such as limited data availability and measurement errors. In this study, manure temperature was measured manually in a limited number of observations (six times over one year). The calculated relative humidity was relatively underestimated by the model compared to the continuous measured data (MAE and RMSE > 10% and R^2^ of 0.57 and 0.63 in both systems).

### 3.2. Methane Emission

A summary of the calculated and measured volatile solids (VS) and methane (CH_4_) emission from the LS and SS systems are presented in Table 5. The calculated vs. content of the manure was lower than the measured value (ca. 10% with MAE of around 2.6 g kg^−1^ RMSE in the range of 11.2–12 g kg^−1^ and average R^2^ of 0.20 for both systems). The average calculated CH_4_ emission (18.5 and 4.3 kg yr^−1^ per pig place) was in between the means from the continuous data (15.9 and 5.6 kg yr^−1^ per pig place) and the means from the discrete measurements using the reference method (22.0 and 3.1 kg yr^−1^ per pig place) for LS and SS, respectively. The largest RMSE was observed between the predicted and continuous measurements, with a value of 4.6 kg yr^−1^ per pig place.

**Figure 3:**
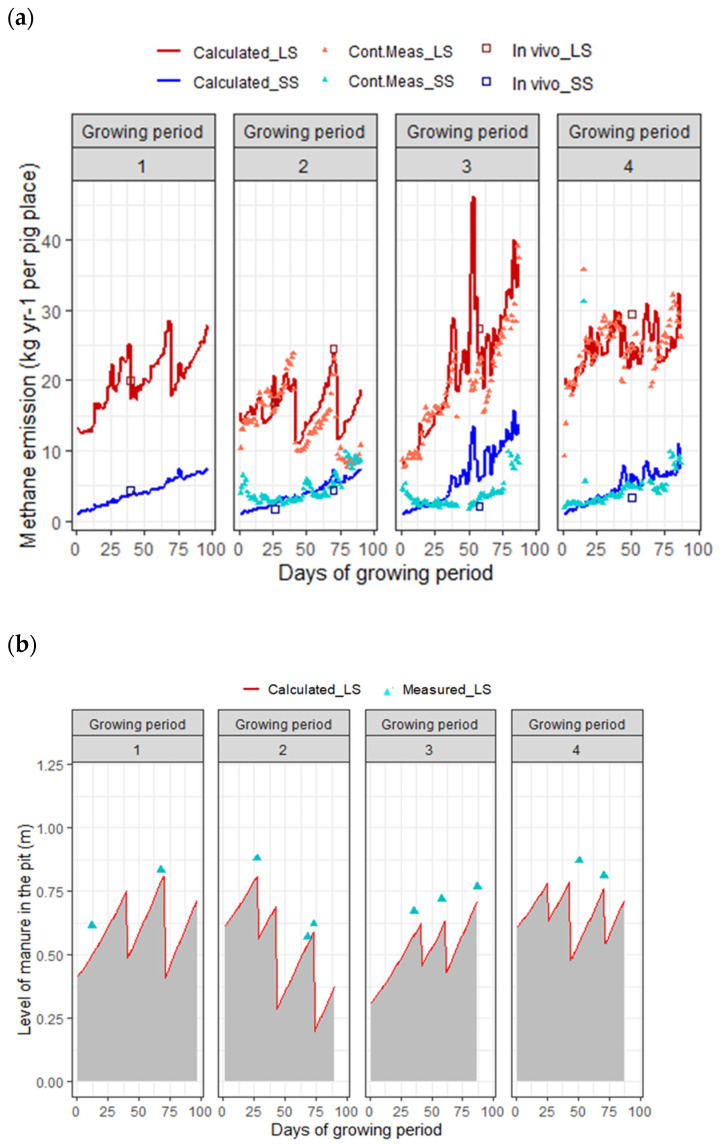
Calculated (line) and measured (point) methane emission (kg yr^−1^ per pig place) (**a**) and height of the manure accumulated in the pit (**b**) per growing period (GP) for two manure management systems (MMS) in fattening pig rooms. LS: long storage in pit with straight walls (the red symbols and line) and SS: short storage in pit with sloped walls (the blue symbols and line). All data represents the mean daily values converted to annual levels. The ‘Cont.Meas’ category indicates the continuous measurements using sensors. The ‘in vivo’ data indicates the discrete reference measurements. In growing period 1, no sensor measurements were recorded. The start and end dates of the growing periods were: GP1: 8 October 2020–12 January 2021; GP2: 21 January 2021–20 April 2021; GP3: 27 April 2021–21 July 2021; GP4: 27 July 2021–21 October 2021.

The development of calculated and measured methane emission and the height of manure in the pit representing the manure volume are presented in Figure 3a,b. Based on this graph, the predicted volume of the stored manure and the corresponding methane emission fitted well with the measurements. The breaks seen in this graph are due to the partial emptying of the pit on certain days during each growing period. No continuous measurement data were available for the first growing period (GP1) due to unreliable sensor calibrations. Based on the results of the in vivo measurements (the reference method) for SS, a reduction potential for methane emission of 86.0% (±5.6%) compared with LS can be expected.

Comparing the measured CH_4_ emission (continuous and point measurements) with the calculated values implies that the model predictions fitted quite well with the measurements. The mean of the measured (reference method) and calculated methane emission of the LS system were 22.0 ± 5.0 and 18.5 ± 5.3 kg yr^−1^ per pig place, respectively. Those for the SS system were 3.1 ± 1.3 and 4.3 ± 2.4 kg yr^−1^ per pig place, respectively. The variation observed between continuous CH_4_ emission measurements obtained from the sensors and discrete measurements using the reference method can be attributed to several factors. The primary factor to note is that the sensors utilized in our experiment were new and had been calibrated by the manufacturer, without specific calibration for our study. Hence, it is reasonable to attribute any discrepancies to potential errors in calibration. The secondary factor: the main objective of sensor measurements was to monitor emission patterns with a lesser focus on measuring absolute emissions. Furthermore, ensuring the proper functioning of the sensors is imperative and requires ongoing monitoring.

During the growing period, methane emission was mainly influenced by the level of manure stored in the pit, featuring lower emission rates after each (partial or full) emptying of the manure pit (Figure 3b). For SS, the manure pit was emptied every day, while the water channel was emptied once per growing period. This partially explains the increasing pattern of methane emission in the SS system (blue lines), besides the expected increase in methane production from enteric fermentation. In the third growing period (GP3), it is likely that the water channel of the SS system was emptied more frequently, in line with farm practices implemented on the commercial farm, though these data have not been registered and therefore have not been accounted for in the model inputs. Consequently, this change may have contributed to the observed reduction in CH_4_ emission, as detected by both the sensor and the reference method, in contrast to the predictions generated by the model.

For a better prediction level, detailed farm records of the removal volume, its frequency, and the residual manure in the pit are necessary inputs to the model. In this study, the height of manure in the deep pit and the level of the water channel were not regularly measured and observed. The model, therefore, assumes a linear correlation between the estimated volume of the manure, estimated by the height and area of the manure pit, and the CH_4_ emission. In SS, it is expected that, with a more frequent emptying system in the water channel and thorough cleaning of the channels after flushing, CH_4_ emission can be reduced to even a larger extent. Due to the daily and (almost) complete removal of the manure, the anaerobic conversion of the organic matter in the manure to methane and carbon dioxide barely occurs [42]. By limiting the growth and activity of methanogenic communities and by reducing the amount of organic matter by the more frequent removal of manure to outside storage, lower methane emission rates can be expected [43]. Another study has recently shown that methane emission is highly dependent on the frequency of manure removal and less dependent on temperature of the manure in pig house storage with increased manure removal frequencies [20]. Therefore, the present findings indicate that daily manure removal can significantly diminish methane emission, to the extent that the only source of CH_4_ is the enteric methane from the pigs. The enteric methane from pigs was reported as approx. 1.5 kg pig^−1^ year^−1^ produced by pigs themselves [44]. Thus, it is to be expected that, with SS, most methane emission from manure could be prevented.

Another general observation is that, in addition to manure management practices, CH_4_ emission in the LS system was influenced by seasonal effects (Figure 3a). Specifically, the average methane emission during the summer months (GP3 and GP4, encompassing June and July) exceeded that of the other two growing periods occurring in the autumn and winter seasons. The current model predicts the manure temperature based on the room temperature, and the room temperature is calculated according to the outside temperature. We stress, therefore, the importance of accurately predicting the manure temperature in relation to the manure volume in the storage for accurate prediction of CH_4_ emission.

**Figure 4:**
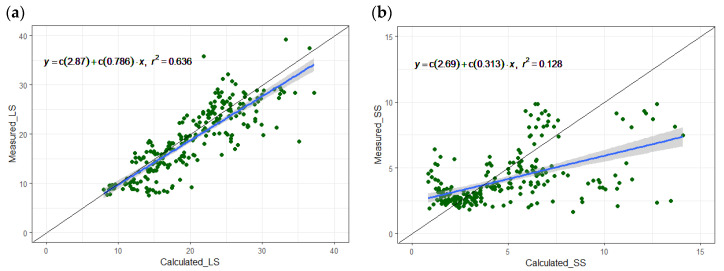
The continuous-measured (Y) and calculated (X) methane emission (kg yr^−1^ per pig place) in (**a**) LS: long storage in pit with straight walls; (**b**) SS: short storage in pit with sloped walls. The solid black line represents the 1:1 line. The points represent the measured data points and the blue line is the regression line between (X) and (Y) showing the best-fit line throughout the data points. All data represents the mean daily values converted to annual emission.

The linear relationships between the continuous-measured and calculated methane emission are shown in Figure 4. The results show that the model has a better prediction for LS than SS (R^2^ of ca. 64% and 13%). This difference in prediction can be explained by the higher number of assumptions about the LS system (for example, the cleaning level of the manure pit, as well as the emptying intervals of the water channel, which may not be fully aligned with the practical situation of the examined farm). By removing the predicted values of the third growing period (GP3), the R^2^ increases (by a factor of 2). Overall, improving the inputs of the model with accurate activity data from farm practices can increase the accuracy of the prediction.

### 3.3. Ammonia Emission

A summary of the calculated and measured ammonia emission per source (manure pit and soiled floor surface) for one year are presented in Table 6. The average values of most of the measured parameters were comparable with the calculated values. The mean calculated ammonia emission for LS and SS were 2.6 (±1.0) and 1.4 (±0.8) kg yr^−1^ per pig place, respectively. The calculated and measured parameters were in good agreement, confirming the accuracy of the model predictions. The MAE of NH_3_ emission was 0.8 and 0.5 kg yr^−1^ per pig place. Additionally, the RMSE was 1 and 0.5 kg yr^−1^ per pig place with an R^2^ of 0.45 and 0.67 for the LS and SS systems, respectively. From these results, it is also evident that the NH_3_ emission emitted from the manure pit is higher than from the floor (on average, 80% for LS and 70% for SS were released from the manure pit). This highlights the impact of mitigation measures on reducing NH_3_ emission from the inside storage pits compared to the floor. Measures controlling the indoor climate of the pig houses and management factors affect ammonia formation on the floor surface. For example, in pig houses, the risk of soiling becomes greater as the areaof solid floor increases. This risk can be limited by a good pen design and by maintaining a good indoor climate (e.g., by air or floor cooling in the summer) [34]. Many studies have shown that lower ammonia emission can be achieved with partly slatted floors, provided that the solid part of the floor remains clean [45,46,47].

**Figure 5:**
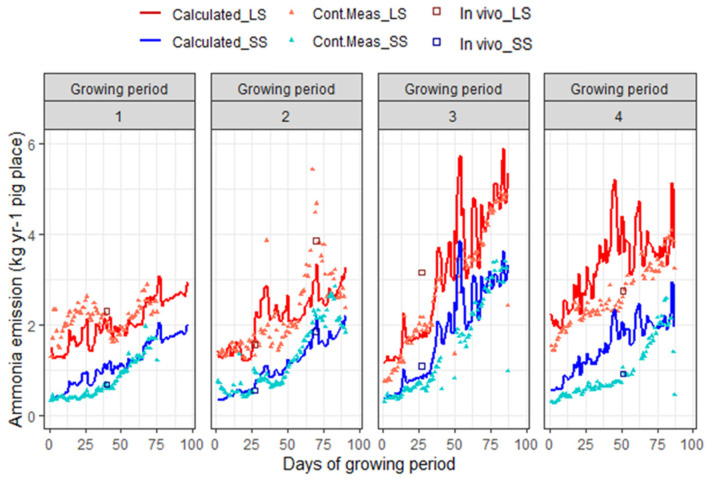
Calculated (line) and measured (point) ammonia emission (kg yr^−1^ per pig place) for two manure management systems (MMS); LS: long storage in pit with straight walls (the red symbols and line); SS: short storage in pit with sloped walls (the blue symbols and line). Category ‘Cont.Meas’ indicates the continuous measurements using sensors. Category ‘in vivo’ indicates the discrete reference measurements. Breaks are due to problems with the sensor or outlier detection. The start and end dates of the growing periods were: GP1: 8 October 2020–12 January 2021; GP2: 21 January 2021–20 April 2021; GP3: 27 April 2021–21 July 2021; GP4: 27 July 2021–21 October 2021.

The total ammonia emission obtained from the model predictions (lines) in comparison with the sensor measurements (triangular points) and the reference method (in square and circle) are represented in Figure 5. This graph also demonstrates the reduction potential in ammonia emission from the manure pit affected by the manure management system and pen design (the red line and points for LS and the blue ones for SS). The average ammonia emission reduction measured by the reference method corresponds to 62.7% (±7.4%), with an average emission rate of 2.63 and 1.0 kg yr^−1^ per pig place (Booijen et al., 2023) [41]. The discrepancy observed between continuous NH_3_ emission measurements obtained from the sensors and discrete measurements using the reference method can be attributed to several factors, as mentioned above. The new sensors, utilized in this study at the commercial farm, were not calibrated within this study. Additionally, potential inaccuracies in the NH_3_ sensors, particularly in measuring very low (<2 ppm) and very high concentrations, may contribute to the variation. During the last two growing periods (GP3 and GP4), the predicted NH_3_ emission surpassed both the continuously measured data and reference measurements. This disparity could be linked to the predicted ambient and manure surface temperatures during the warm season.

From the results, it can be concluded that the prediction of ammonia emission was well-aligned and reasonably accurate for both systems when compared with the reference points. Overall, the reduction measures in the SS system, including reduction of the slurry pit surface with sloped pit walls, frequent manure removal, and dilution with water, demonstrated relatively the good reduction potential of NH_3_ emission compared to the reference system.

**Figure 6:**
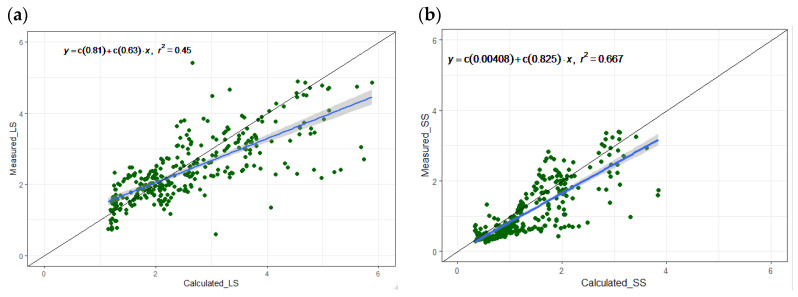
The measured (Y) and calculated (X) ammonia emission (kg yr^−1^ per pig place) of two MMS (**a**) LS: long storage in pit with straight walls; (**b**) SS: short storage in pit with sloped walls. The solid black line represents the 1:1 line. The points represent the measured data points and the blue line is the regression line between (X) and (Y) showing the best-fit line throughout the data points. Data represents the average daily values converted to annual level.

The linear relationships between the daily measured and calculated ammonia emission per MMS are shown in Figure 6. The slopes of the linear regression line and *R*^2^ were around 0.63 and 0.45 for LS and 0.83 and 0.67 for SS. This relationship also suggests that as emission rates increase, the variability in emission becomes higher, leading to lower prediction accuracy.

### 3.4. Model Parameters and Implications for the Predicted Emission

A model approach in estimating the influence of mitigation measures has been introduced in this study. The most critical component in these predictions is water excretion, due to the error caused by the incorrect estimation of water evaporation from manure and fouled floors with urine, which is affected by the air velocity and temperature at the surface. Within the model, the air velocity above the evaporation surface is assumed to be constant.

Model predictions can be improved by better prediction of the pH of the manure. The current model lacks an adequate prediction of manure pH, which is a crucial factor influencing NH_3_ emission. This is partly due to the fact that a large number of factors are affecting the pH; in particular, the carbonate content of urine and manure is hard to predict. As an intermediate solution, the pH of the urine and manure was measured and used as an input for the model. The pH of the top layer of the manure was determined from the pH of the bulk of the manure based on a lab-scale analysis at the University of Southern Denmark in Odense [34]. Further development of the model should focus on the accurate estimation of the pH by using a measurement set-up to measure the surface pH of the top 0.1 mm of manure, comparable to a practical situation, or using the measured pH of the bulk manure as an input for the emission model.

Another important point for improvement of the model is the assumption about the emitting surface. In this current version of the model, the ammonia emission per m^2^ of contaminated concrete slatted floor was assumed to be the same as that of the concrete solid floor. This current model could be further improved by developing a dynamic urination model for determining the variation in ammonia emission over time (on an hourly basis), as suggested by [33].

Temperature and air velocity above the emitting surface were estimated from the measured temperature and ventilation quantity at the exhaust, which, although currently easily measured, can be nevertheless improved by air flow models to better estimate the temperature and air velocity above different emitting surfaces. Extra effort is required to incorporate and apply these features into this model in a simple way. Further development of the methane emission model should be focused on temperature variation by the depth at inside storages and degradability of the organic matter over the storage period. Furthermore, the accuracy of the model is heavily reliant on precise and adequate input data. In this work, the regular recording of the manure level height in the storage was lacking. It is anticipated that with a larger dataset, the predicted methane emission will be improved.

It should be pointed out that the model currently uses the temperature-related equation for the estimation of methane emission. This relationship is dependent on the *lnA* value [32]. The *lnA* value used in this study was obtained from the study by [32,37]. Therefore, for a better estimation of methane emission using the approach used in this study, it is recommended to parameterize the model by the determination of *lnA* for country-specific and system-specific (linked to various manure management systems) pig farms in the Netherlands.

## 4. Conclusions

This paper discussed the prediction of CH_4_ and NH_3_ emissions through a model-based approach. The study compared the results obtained by integrating two comprehensive models (ANIPRO + MESPRO) with values derived from continuous data measurements and a discrete reference method. The model approach was applied to two fattening pig houses implementing emission reduction measures and compared with a conventional housing system for pigs in the Netherlands. The main conclusions drawn from this study are as follows:

1. The average calculated CH_4_ and NH_3_ emissions on an annual basis correspond well with measured values for the examined measures.
2. The measurements confirmed the reduction potential of the studied measures for CH_4_ and NH_3_ emissions from pig houses. The model could predict these effects with an acceptable degree of accuracy.
3. The obtained results suggest that improving the calculation rules of the model for better estimation of variables affecting ammonia emission, such as the pH, temperature, and air velocity, will lead to a better prediction of emissions.
4. The model attributes provide valuable means for assessing the impact of mitigation measures on CH_4_ and NH_3_ emissions. This provides a robust basis for assessing the impact of management and housing strategies on CH_4_ and NH_3_ emissions from pig houses, which, in turn, helps support more sustainable practices in pig farming.

## Figures and Tables

**Table 1:** Estimated parameters of the Gompertz curve (see Equation (1)), by regression on measured data, for calculating weight, feed, and drinking water intake of fattening pigs. The standard deviations are shown in brackets.

Parameter ^1^	Weight	Feed Intake	Drinking Water Intake
*A*	0 ^2^	−41.9 (13.1)	−149 (108)
*W_m_*/FI_m_/DWI_m_ ^3, 4^	164.2 (3.9)	608 (48.3)	1432 (115.1)
*B*	0.0146 (0.0010)	0.0111 (0.0017)	0.0103 (0.0042)
t* ^5^	110.4 (4.3)	154.7 (10.8)	147.4 (23.9)

^1^ For an explanation of the variables, see Equation (1). ^2^ The constant was set to zero, because including it in the regression formula did not provide a better fit. ^3^ FI_m_ and DWI_m_ are the parameters for feed and drinking water intake, comparable to *W_m_* for the growth curve. ^4^ These values are fitted in the model to the input data (the initial and final weight of the pigs corresponding to *W_m_*, and the total feed and water intakes corresponding to FI_m_ and DWI_m_, respectively). ^5^ t* is the time when growth is maximal (day).

**Table 2:** Key parameters of the methane estimation model, based on previous studies (*E_a_* and *lnA*) and this study (*VS_d_*).

Parameter	Petersen et al. (2016) [32]	This Study
*VS_d_* (kg kg^−1^ VS)	0.51	0.83
*E_a_* (kJ mol^−1^)	81.0 *	81.0 *
*lnA* (g CH_4_ kg^−1^ vs. h^−1^)	31.3	31.3 **

* adopted from [37]; ** adopted from [32]; *VS_d_*: degradable volatile solids; *E_a_*: Apparent activation energy (kJ mol^−1^); *A*: the Arrhenius parameter.

**Table 3:** Overview of the experimental pig rooms with two different manure management systems.

Characteristics	LS ^1^	SS ^2^
No. of animal places	54	78
Growth range (kg)	23.6–115.6	22.6–114.0
Room length (m) × width (m)	11.28 × 5.90	15.55 × 6.00
No. of pens	6	6
Pen length (m) × width (m)	5.10 × 1.88	5.22 × 2.59
Depth of manure pit	1.20	0.50
Area per animal (m^2^ pig^−1^)	1.00	1.00
Material slatted floor (back-front slatted floor)	Metal triangular—Concrete	Metal triangular—Concrete
Material solid floor	Concrete	Concrete
Slatted floor/Solid floor (%)	60/40	38/62
Slope of manure pit wall (°)	90	45
Manure removal interval (d)	45 ^3^	1
Feeding/Drinking system	Dry feeder/Nipple	Dry feeder/Nipple

^1^ LS: long term storage of manure; ^2^ SS: Short term storage; ^3^ Mean of emptying interval.

**Table 4:** Average calculated and measured room temperature, manure temperature, and relative humidity for two manure management systems (MMS) in fattening pig rooms. Standard deviations are given between parentheses.

Variable	MMS	Calculated (Model)	Measured (Continuous)	Measured (Discrete) ^3^	MAE ^4^	RMSE ^5^	R^2^
Room temperature (°C)	LS ^1^	22.3 (1.6)	23.0 (1.8)	-	1.5	4.0	0.80
	SS ^2^	21.4 (2.7)	21.0 (2.6)	-	1.1	3.8	0.92
Manure temperature (°C)	LS	19.5 (1.3)	-	24.5 (1.7)	6.7	6.5	<1
	SS	18.7 (2.2)	-	22.3 (2.4)	6.8	7.5	<1
Relative humidity (%)	LS	59.7 (4.1)	67.6 (4.4)	-	9.4	13.1	0.57
	SS	56.1 (5.3)	67.9 (5.0)	-	13.1	15.5	0.63

^1^ LS: long storage system; ^2^ SS: Short storage system; ^3^ Reference method [40]; ^4^ Mean absolute error; ^5^ Root mean square error between calculated and measured values based on daily differences.

**Table 5:** Average calculated and measured volatile solids and methane emission for two manure management systems (MMS) in fattening pig rooms. Standard deviations are given between parentheses.

Variable	MMS	Calculated (Model)	Measured (Continuous)	Measured (Discrete) ^3^	MAE ^4^	RMSE ^5^	R^2^
Volatile solids-manure (g kg^−1^)	LS ^1^	68.5 (5.9)	-	76.2 (11.5)	2.6	12.5	0.16
	SS ^2^	68.0 (7.3)	-	77.4 (9.0)	2.8	11.2	0.21
CH_4_ emission(kg yr^−1^ per pig place)	LS	18.5 (5.3)	15.9 (7.9)	22.0 (5.0)	3.1	4.6/2.8	0.64
	SS	4.3 (2.4)	5.6 (3.7)	3.1 (1.3)	1.9	3.3/3.0	0.13

^1^ LS: long storage system; ^2^ SS: Short storage system; ^3^ Reference method [40]; ^4^ Mean absolute error; ^5^ Root mean square error between calculated and measured (continuous/discrete) values based on daily differences.

**Table 6:** Average calculated and measured ammonia emission sources for two manure management systems (MMS) in fattening pig rooms. Standard deviations are given between parentheses.

Variable	MMS	Calculated- Model	Measured - Continuous	Measured - Discrete ^3^	MAE ^4^	RMSE ^5^	R^2^
NH_3_ emission—Floor (kg yr^−1^ per pig place)	LS ^1^	0.5 (0.3)	-	-	-	-	-
	SS ^2^	0.5 (0.3)	-	-	-	-	-
NH_3_ emission—Manure pit (kg yr^−1^ per pig place)	LS	2.2 (0.9)	-	-	-	-	-
	SS	0.9 (0.6)	-	-	-	-	-
Total NH_3_ emission (kg yr^−1^ per pig place)	LS	2.6 (1.0)	2.6 (0.9)	2.71 (0.4)	0.8	1.2/1.1	0.45
	SS	1.4 (0.7)	1.2 (0.9)	1.01 (0.2)	0.5	0.8/0.3	0.67

^1^ LS: long storage system; ^2^ SS: Short storage system; ^3^ Reference method [40]; ^4^ Mean absolute error; ^5^ Root mean square error between calculated and measured (continuous/discrete) values based on daily differences.

## Data Availability

The data presented in this study are available on request from the corresponding author. The data are not publicly available due to the data still being processed to produce other papers.

## Supplementary Materials

The following supporting information can be downloaded at https://www.mdpi.com/article/10.3390/ani14060964/s1. The supporting material is published online alongside this paper, including ädditional equations and a figure of the result section (Figure S1 Calculated (line) and continuous-measured (point) values of indoor temperature (°C), relative humidity (%) and ventilation rate (m^3^ h^−1^ per pig place). The red lines and points represent the reference department and those in blue represent the trial department. Each segment indicate one growing period (GP); 8 October 2020–12 January 2021; GP2: 21 January 2021–20 April 2021; GP3: 27 April 2021–21 July 2021; GP4: 27 July 2021–21 October 2021.) [32,33,34,48,49,50,51,52].
