# Supplementary material for: Model Adaptation and Validation for Estimating Methane and Ammonia Emissions from Fattening Pig Houses: Effect of Manure Management System
Source: Animals (Basel). 2024 Mar 20;14(6):964. doi: 10.3390/ani14060964 (PMC10967431; doi:10.3390/ani14060964)
Supplement: Supplementary file 1 [file animals-14-00964-s001.zip › animals-2825217-supplementary.pdf]

Supplementary Materials

Model Adaptation and Validation for Estimating Methane and Ammonia Emissions from Fattening Pig Houses: Effect of Manure Management System

Paria Sefeedpari *, Seyyed Hassan Pishgar-Komleh and Andre J. A. Aarnink

Wageningen Livestock Research, Wageningen University and Research, P.O. Box 135, 6700 AC Wageningen, The Netherlands; hassan.pishgarkomleh@wur.nl (S.H.P.-K.); andre.aarnink@wur.nl (A.J.A.A.)
* Correspondence: paria.sefeedpari@wur.nl

1. Ammonia emission

The different variables and constant values of ammonia model (Eq. 2 of the main manuscript) are calculated as follows (Aarnink and Elzing, 1998) [33]:

$$k_{NH3} = 50.1 \cdot (v_{surface})^{0.8} \cdot (T_{surface})^{-1.4}$$ Eq. S1

where,

k_{NH3} is the mass transfer coefficient for ammonia (m/s)

$v_{surface}$ is the air speed over the emissive surface (m/s)

$T_{surface}$ is the temperature of the emissive surface (K).

$$f = \frac{10^{pH_{surface}}}{10^{pH_{surface}} + 5 \cdot 10^{(0.0897 + (\frac{2729}{T_{surface}})}}$$ Eq. S2

where,

f is the fraction of unionized ammonia in the solution (-)

$pH_{surface}$ is the pH of the emissive liquid at the surface (-)

$T_{surface}$ is the temperature of the emissive surface (K).

$$H = 1431 \cdot 1.053^{(293 - T_{surface})}$$ Eq. S3

where,

H is Henry's constant

$T_{surface}$ is the temperature of the emissive surface (K).

The NH_4-N content of the bulk slurry was calculated by assuming complete conversion of the urea in urine to NH_4- N. Knowing the total amount of excreted urea-N, the NH_4-N concentration of the bulk slurry can be calculated.

The potential excretion of NH_4-N (total of urea-N and NH_4-N) in fresh urine is calculated from the total N excretion in the fresh urine (other N compounds make up the rest of the N):

$$U_{NH4N-pot} = -2{,}7 \cdot 10^{-4} + 0{,}87 \cdot U_{Ntot} \quad \text{Eq. S4}$$

where,

$U_{NH4N-pot}$ is the potential NH_4-N excreted per day (total of NH_4-N and urea-N in urine) (kg/d),

U_{Ntot} is the total amount of N excreted in urine (kg/d).

Measurements have revealed that in fattening pigs, approximately 11.0% (standard error (se): 0.3%) of the faeces-N exists in the form of NH_4-N. It is anticipated that this NH_4-N will not, or will only minimally, emit from the floor. Nevertheless, it remains crucial for determining the NH_4-N content of the slurry (mixed urine and faeces) in the manure pit [34].

The air velocity over the manure surface in the manure pit was calculated using the following relationship with the ventilation flow rate per square meter (m^2) of barn area, derived from the best fit of the measured data.

$$v_{surface\_pit} = 0{,}091 + 14{,}2 \cdot vent_{m^2} \quad \text{Eq. S5}$$

where,

$v_{surface\_pit}$ is the air velocity over the evaporating surface (m/s),

$vent_{m2}$ is the ventilation flow per m^2 of barn area (m^3/s per m^2 of barn area).

Measurements have shown no significant correlation between the air velocity measured above the urine-soiled floor surfaces and the ventilation flow rate per m^2 of the barn area. Therefore, the air velocity is assumed to remain constant above the urine soiled area (0.146 m/s). Furthermore, the air velocity and the temperature of the emitting surface is assumed to be equal to that of the manure in the pit. The following relationship was found between the temperature of the emitting surface in the manure pit and the temperature of the outgoing air from the pig house:

$$T_{surface\_manure} = 1{,}85 + 0{,}735 \cdot T_{exhaust}$$ Eq. S6

where,

$T_{surface\_manure}$ is the temperature of the emitting manure surface in the basement (°C),

$T_{exhaust}$ is the temperature of the outgoing stable air (°C).

This regression line accounts for 42% of the variation in the measured surface temperature of the manure pit (Aarnink *et al.*, 2018)[34]. The surface temperature of the urine samples was comparable to the outgoing air temperature (regression coefficient equal to 1.01; se 0.02%) and was therefore set equal to this temperature in the model.
The water evaporation from the soiled floor surfaces and the manure pit has been found to be related to the vapor pressure difference in the interface of the wet surface and the air and is proportional to the size of it evaporating surface. The evaporation from the wet floor surfaces and the manure pit was estimated as follows:

$$evap_{floor} = k\_evap_{floor} \cdot (p_{floor} - p_{air}) \cdot A_{wet-floor}$$ Eq. S7

$$evap_{pit} = k\_evap_{pit} \cdot (p_{pit} - p_{air}) \cdot A_{wet-pit}$$ Eq. S8

where,

$evap_{floor}$ / $evap_{pit}$ is the water evaporation from the floor or the manure pit (kg/d),

k_evap_{floor} / $k\text{-}evap_{pit}$ is the evaporation coefficient of the floor or the manure pit (kg/(m^2.kPa.d)),

p_{floor} / p_{pit} is vapor pressure of the wet floor surface or the pit surface (kPa),

p_{air} is the vapor pressure of the air (kPa).

It is assumed here that the relative humidity of the evaporating surface of the floor and manure cellar is 100%. The vapor pressures have been calculated using Albright (1990) formulas [48]. According to Beeking *et al.* (1994) [49] is the evaporation coefficient proportional to the square root of the air velocity over the evaporating surface:

$$k_{evap} = b_{k_{evap}} \cdot \sqrt{v_{surface}}$$ Eq. S9

where,

k_{evap} is the evaporation coefficient for the floor or manure pit ($kg/(m^2.kPa.d)$)

b_{kevap} is a regression coefficient,

$v_{surface}$ is the air speed over the evaporating surface (m/s).

The regression coefficient *b* is based on the least squares method on the measured evaporation data. This analysis led to estimation of b_{kevap} as 7.69 (Aarnink *et al.*, 2018)[34].

2. Methane emission from manure pit

In this model, volatile solid was considered as a main determining factor for CH_4 emissions from animal manure storages divided into degradable (VS_d) and non-degradable VS (VS_{nd}) (Sommer et al., 2004). In Eq. 3, the model assumes that the degradation of VS_{nd} is 100-fold slower than the degradation of VS_d. Furthermore, the VS degradability in the manure pit is corrected for the lignin content of the diet (assuming that lignin is non-degradable) and subsequently the fractions of degradable VS were calculated. The lignin content (%) as a percent of total solids is identified with Acid Detergent Lignin (ADL) (assuming that the fraction of lignin-bound nitrogen is insignificant) (Soest, 1963; Triolo et al., 2011) [50,51]. The ADL content of feed ingredients were derived from CVB tables (2021) and calculated for the studied diets. The content of volatile solids of the faeces and urine are predicted by the model. The organic matter excretion in faeces was estimated based on the indigestible organic matter of the feed intake (Rigolot *et al.*, 2010) [52]. Thus the total VS content was calculated using Eq. S10 – S13.

$$F_{vs} = FI \times Feed_{OM} \times (1 - d_{COM}) \quad \text{Eq. S10}$$

$$U_{vs} = \frac{60}{28} \cdot U_{Ntot} \quad \text{Eq. S11}$$

$$VS_{total} = F_{vs} + U_{vs} \quad \text{Eq. S12}$$

$$VS = \frac{VS_{total}}{total\ manure\ excretion} \quad \text{Eq. S13}$$

where,

F_{vs} is the organic matter excretion via faeces (kg/d),

FI is the feed intake (kg/d),

$Feed_{OM}$ is the organic matter of the feed, input in the model (kg/kg),

d_{COM} is the digestibility coefficient of the organic matter of the diet, input in the model (kg/kg),

U_{vs} is the organic matter excretion via urine (kg/d),

VS_{total} is the total organic matter excretion (kg/d),

VS is the volatile solids content of the manure (kg/kg).

4.1 Temperature correction

The CH_4 production rate was measured following a measurement protocol (Petersen et al., 2016) [32] under an average temperature of all samples while the *in-situ* temperature of manure (measured during the measurement campaign on the site) might differ (according to the protocol of this study the difference should not exceed ±3 °C); Therefore the measured rates were corrected to the exact temperature of the manure recorded at the time of sampling. The corrected methane production rate for temperature can be calculated by Eq. S14.

$$k_2 = k_1\, exp\, (\frac{-E_a}{R}\left(\frac{1}{T_2} - \frac{1}{T_1}\right)) \qquad \text{Eq. S14}$$

where,

k_2 is the rate at in-situ temperature (g CH_4 kg^{-1} VS d^{-1}),

k_1 is the rate measured (g CH_4 kg^{-1} VS d^{-1}),

T_1 is the incubation temperature (K),

T_2 is the measured in-situ temperature (K) of the manure sample.

(a) (b)

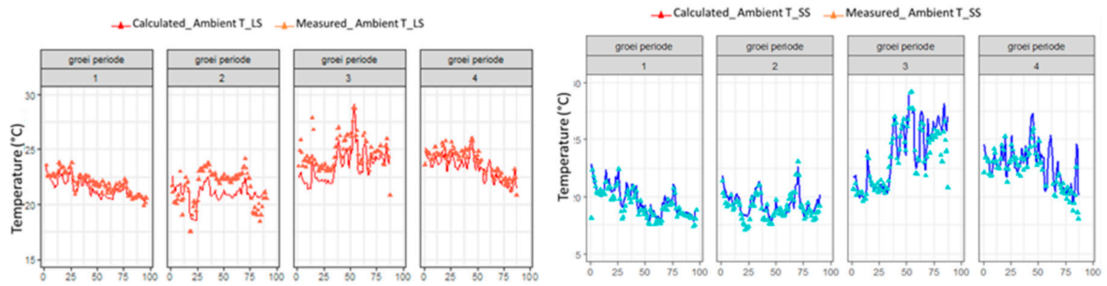

(d)

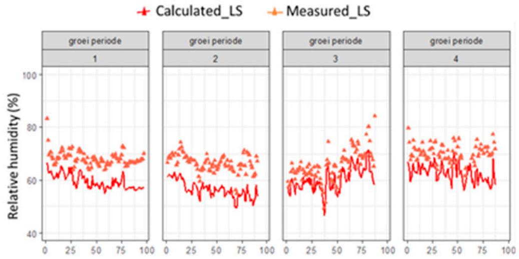

(c)

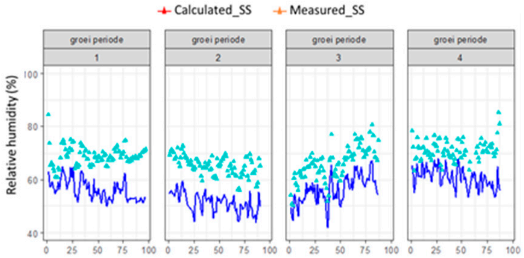

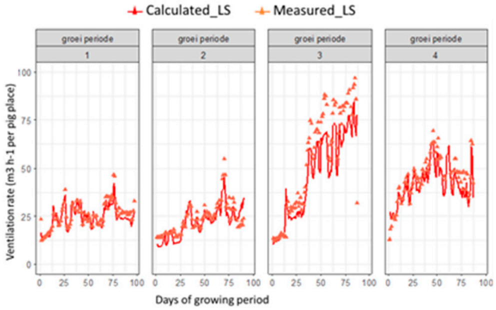

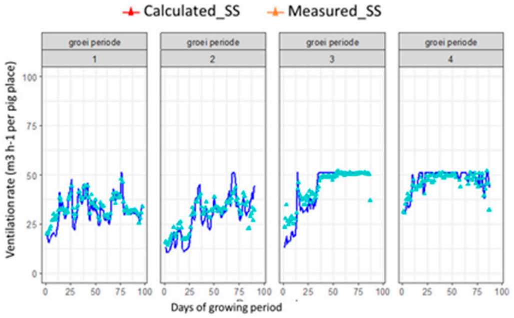

(e)

(f)

Figure S1 Calculated (line) and continuous-measured (point) values of indoor temperature (°C), relative humidity (%) and ventilation rate (m^3 h^{-1} per pig place). The red lines and points represent the reference department and those in blue represent the trial department. Each segment indicate one growing period (GP); 8 October 2020–12 January 2021; GP2: 21 January 2021 – 20 April 2021; GP3: 27 April 2021 – 21 July 2021; GP4: 27 July 2021 – 21 October 2021.

References

32. Petersen, S.O., Olsen, A.B., Elsgaard, L., Triolo, J.M., Sommer, S.G., 2016. Estimation of methane emissions from slurry pits below pig and cattle confinements. PloS one 11, e0160968.

33. Aarnink, A.J.A., Elzing, A., 1998. Dynamic model for ammonia volatilization in housing with partially slatted floors, for fattening pigs. Livest. Prod. Sci. 53: 153-169.

34. Aarnink, A.J.A., Van de Pas, P.A., Van der Peet-Schwering, C.M.C., Hol, A., Binnendijk, G.P., Le Dinh, P., Hafner, S.D., Ogink, N.W.M., 2018. Rekentool voor het bepalen van de effecten van voer- en management-maatregelen op de ammoniakemissie bij varkens: ontwikkeling en validatie [Calculation tool to estimate the effects of feeding and management measures on ammonia emission in pigs]. Wageningen Livestock Research, Report 1086, Wageningen, p. 118.

48. Albright, L.D., 1990. Environment control for animals and plants. American Society of Agricultural Engineers.

49. Beeking, F., Ingelaat, F.B., van Beek, G., 1994. De specifieke vochtafgifte van leghennenmest. COVP-DLO.

50. Soest, P.V., 1963. Use of detergents in the analysis of fibrous feeds. II. A rapid method for the determination of fiber and lignin. Journal of the Association of official Agricultural Chemists 46, 829-835.

29. Sommer, S., Petersen, S., Møller, H., 2004. Algorithms for calculating methane and nitrous oxide emissions from manure management. Nutrient Cycling in Agroecosystems 69, 143-154.

51. Triolo, J.M., Sommer, S.G., Møller, H.B., Weisbjerg, M.R., Jiang, X.Y., 2011. A new algorithm to characterize biodegradability of biomass during anaerobic digestion: influence of lignin concentration on methane production potential. Bioresource technology 102, 9395-9402.

52. Rigolot, C., Espagnol, S., Robin, P., Hassouna, M., Béline, F., Paillat, J.-M., Dourmad, J.-Y., 2010. Modelling of manure production by pigs and NH3, N2O and CH4 emissions. Part II: effect of animal housing, manure storage and treatment practices. Animal 4, 1413-1424.
